# Reaction of Chromium(III) with 3,4-Dihydroxybenzoic Acid: Kinetics and Mechanism in Weak Acidic Aqueous Solutions

**DOI:** 10.1155/2008/212461

**Published:** 2009-02-04

**Authors:** Kimon Zavitsanos, Konstantinos Tampouris, Athinoula L. Petrou

**Affiliations:** Laboratory of Inorganic Chemistry, Department of Chemistry, University of Athens, Panepistimioupolis, 15771 Athens, Greece

## Abstract

The interactions between chromium(III) and 3,4-dihydroxybenzoic acid (3,4-DHBA) were studied resulting in the formation of oxygen-bonded complexes upon substitution of water molecules in the chromium(III) coordination sphere. The experimental results show that the reaction takes place in at least three stages, involving various intermediates. The first stage was found to be linearly dependent on ligand concentration *k*_1(obs)_′ = *k*_0_ + *k*_1(obs)_[3, 4-DHBA], and the corresponding activation parameters were calculated as follows: Δ*H*_1(obs)_^*≠*^ = 51.2 ± 11.5 kJ mol^−1^, Δ*S*_1(obs)_^*≠*^ = −97.3 ± 28.9 J mol^−1^ K^−1^ (composite activation parameters) . The second and third stages, which are kinetically indistinguishable, do not depend on the concentrations of ligand and chromium(III), accounting for isomerization and chelation processes, respectively. The corresponding activation parameters are Δ*H*_2(obs)_^*≠*^ = 44.5 ± 5.0 kJ mol^−1^, Δ*S*_2(obs)_^*≠*^ = −175.8 ± 70.3 J mol^−1^ K^−1^. The observed stages are proposed to proceed via interchange dissociative (*I*_*d*_, first stage) and associative (second and third stages) mechanisms. The reactions are accompanied by proton release, as is shown by the pH decrease.

## 1. INTRODUCTION

**Figure 1:**
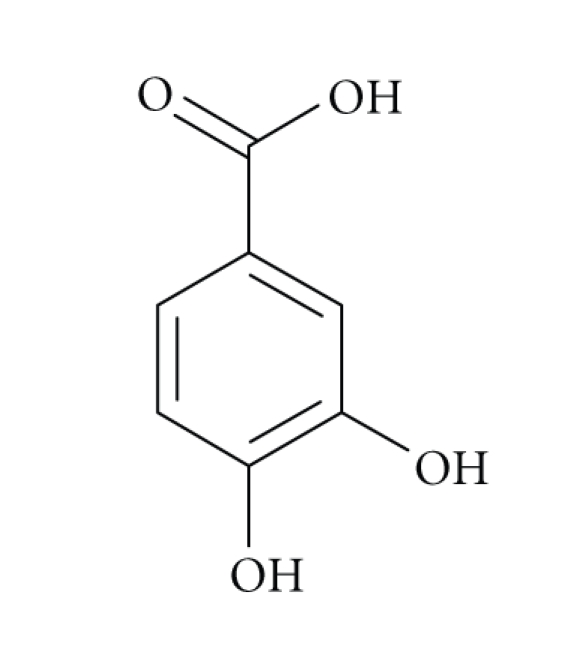
3,4-DHBA.

The ligand 3,4-dihydroxybenzoic acid (3,4-DHBA) (Figure 1), **1**, is known to be produced in the reaction of radicals, which are formed in pathophysiological cases (e.g., ischemic stroke [1, 2], traumatic brain injury [3], and Huntington's disease [4]), the 4-hydroxybenzoic acid or salicylic acid acting as radical-trapping agent [5]. It is used for assisting the Fenton reaction in effluent treatment [6] and in dechlorination of polychlorinated dioxins [7].

3,4-DHBA is employed in the preparation of resins having antioxidant properties, acting as radical scavenger [8] as well as in the preparation of composite polymer modified electrodes [9]. It was also found to interfere in the nucleation and crystal growth of radial alumina trihydrate particles [10].

Phenolic acids, in general, are present in fruits and plants and are participating in the chemical structure of humic substances, which can coordinate with nutrient ions, especially metal ions, increasing thus their bioavailability [11–14]. 3,4-dihydroxybenzoic acid, in particular, bearing both catecholic and carboxylic sites, shows special complexing properties.

Cr(III), although present in traces in biological systems, is considered to play a role in the activation of some enzymes [15]. It has also been identified as a reactive component of an oligopeptide known as low-molecular-weight chromium binding substance or chromoduline [16, 17]. Yet, the biological role of Cr(III) remains mostly unclarified, one of the main reasons being the lack of intense characteristics like charge-transfer bands in the spectra with the only exception being the organochromium complexes [18, 19].

In the present study, the reaction of Cr(III) and 3,4-DHBA in weak acidic aqueous solutions is investigated. The mechanism of the complex formation and the stability of the complexes formed are studied and presented, in order to contribute in the clarification of the role of complexation in the uptake of metals in various biological systems (e.g., plants).

## 2. EXPERIMENTAL RESULTS

### 2.1. Reagents and materials

All reagents employed were of analytical grade and were used as received. Aqueous solutions containing 3,4-DHBA (Alfa Aesar) in concentrations ranging from 7.45 × 10^−3^ to 1.62 × 10^−2^ M were prepared using dilute (0.1 M) KOH solutions for pH adjustment in order for the ligand to be dissolved. Stock solutions (0.2 M) of Cr(III) were prepared from Cr(NO_3_)_2_·9H_2_O. The ionic strength was adjusted using KNO_3_. All solutions used in the present study were freshly prepared in order to avoid side reactions (transformation and decomposition). The addition of the Cr(III) solution kept the pH below 4 due to its acidic hydrolysis.

### 2.2. Kinetic experiments

Electronic spectra were recorded on a Varian Cary 3E, UV-vis spectrophotometer. The kinetic experiments were also followed at the above instrument. All kinetic experiments were performed at pH values below 4 in the presence of air.

**Figure 2:**
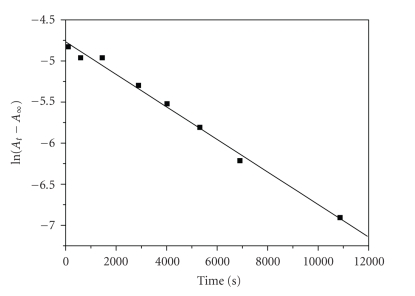
Typical linear plot of ln(*A*_*t*_ − *A*_∞_) versus time at 279 K and [3,4-DHBA] = 1.50 × 10^−2^ M, [Cr(III)] = 2.50 × 10^−3^ M, I = 0.02 M.

Pseudo-first-order conditions were employed for most of the kinetic experiments. For the first stage of the reactions which was studied at 279–295 K, the duration of the step was ~16 000–5000 seconds, the concentrations were of the order of 10^−2^ M for the ligand and 10^−3^ M for Cr(III), and plots of ln(*A*_*t*_ − *A*_∞_) against time (the absorbance is decreasing) were set up, where *A*_*t*_ and *A*_∞_ are absorbances at time *t* and at infinite time (after the completion of this step). The plots were found to be linear (Figure 2) for at least three half lives. The rate constants were calculated from the constant slope of the line. At the higher temperatures where the second and third stages were studied, the first stage being faster is not observed. For the second and third (consecutive) stages which were studied at 303–323K, the duration of the steps was ~20 000–8000 seconds, the concentrations were of the order of 10^−2^ M for the ligand and 10^−3^ M for Cr(III), and plots of ln(*A*_∞_ − *A*_*t*_) against time (the absorbance is increasing) were also found to be linear. This is the case of a polyfunctional compound acting sequentially; a linear plot of ln(*A*_∞_ − *A*_*t*_) = *f *(*t*) could be obtained when a biphasic reaction takes place resembling to a single-stage reaction [20]. This could also happen when *k*_3_ ≫ *k*_2_ or *k*_2_ ≫ *k*_3_. In the case of our system, *k*_2_ could be a lot smaller than *k*_3_. The *k*_1_ and *k*_2_ (*k*_2_ ≪ *k*_3_) values at various temperatures are given in Table 1.

The kinetics was followed at various wavelengths yielding identical results though changes in absorbance were in all cases small. Uncomplexed Cr(III) species does not interfere in the (absorbance) measurements since it is included in both *A*_*t*_ and *A*_∞_ and is thus eliminated.

The activation parameters (Δ*H* ^*≠*^ and Δ*S* ^*≠*^) were calculated from the linear Eyring plots according to activated complex theory. The activation parameters Δ*H*_1(obs)_^*≠*^ and Δ*S*_1(obs)_^*≠*^ corresponding to *k*_1(obs)_ and Δ*H*_2(obs)_^*≠*^, Δ*S*_2(obs)_^*≠*^ corresponding to *k*_2_ (*k*_2_ ≪ *k*_3_) were thus estimated and presented in Table 2.

The *A*_∞_ values were obtained from the kinetic measurements and from the *A* = *f * (*t*) plots (at the certain wavelengths) making it possible to check if the reaction was run to completion.

## 3. DISCUSSION

### 3.1. Kinetics and mechanisms

**Figure 3:**
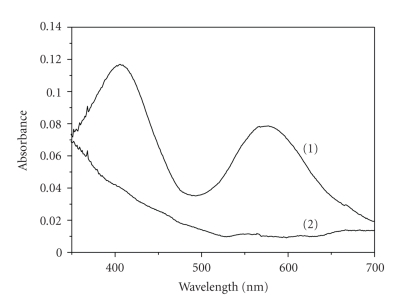
UV-vis spectra of Cr(III) (spectrum 1) and 3,4-DHBA (spectrum 2) solutions of 5.5 × 10^−3^ M concentrations and spectrophotometric cell path *d* = 1 cm.

The UV-vis spectra of Cr(III) and 3,4-DHBA solutions of concentrations 5.5 × 10^−3^ M are presented in Figure 3. At pH < 4, where all the kinetic experiments were conducted, Cr(III) exists mainly in the hexaaqua monomeric form. However, reaction with Cr(H_2_O)_5_(OH)^2+^ should be considered at the pH range 3 to 4, since a small amount of Cr(H_2_O)_5_(OH)^2+^ is present due to the equilibrium which is characterized by a pK_a_ value (Cr^3+^/Cr(OH)^2+^) of about 4 [21]. The visible spectrum of Cr(H_2_O)_6_^3+^ exhibits two maxima in the region of 410 and 575 nm (Figure 3) accounting for the ^4^A_2g_ → ^4^T_1g_ and the ^4^A_2u_ → ^4^T_2g_ Cr(III) transitions, respectively.

Under the applied experimental conditions, the ligand exists as neutral molecule and monoanion [22, 23] abbreviated as DHBA and DHBA^−^, respectively. In the ligand molecules, intramolecular hydrogen bonding between the two hydroxyl groups occurs, favored by the formation of a five-membered ring.

The acid ligand and the Cr(H_2_O)_6_^3+^ complex dissociation equilibria established are as follows:

(1) $$\begin{array}{c} \begin{array}{cc} \text{DHBA} & \overset{{\text{K}}_{1}}{⇌}{\text{DHBA}}^{-}+{\text{H}}^{+}\\ {\text{DHBA}}^{-} & \overset{{\text{K}}_{2}}{⇌}{\text{DHBA}}^{2-}+{\text{H}}^{+}\\ {\text{DHBA}}^{2-} & \overset{{\text{K}}_{3}}{⇌}{\text{DHBA}}^{3-}+{\text{H}}^{+}\\ \text{Cr}{{\left({\text{H}}_{2}\text{O}\right)}_{6}}^{3+} & \overset{{\text{K}}_{\text{a}}}{⇌}\text{Cr}{\left({\text{H}}_{2}\text{O}\right)}_{5}{\left(\text{OH}\right)}^{2+}+{\text{H}}^{+}\;\Delta H_{a},\;\Delta S_{a} \end{array} \end{array}$$

The corresponding pK values for the above acid dissociation constants, at 25°C, are given pK_1_ = 4.5, pK_2_ = 8.7, pK_3_ = 12.8, and pK_a_ = 4.0.

Upon mixing of the reactants, violet Cr(III) and light-brown ligand solutions, formation of a light green complex, **2**, assigned to be oxygen-bound Cr(III) compound takes place. The substitution of water molecules in the Cr(III) coordination sphere by 3,4-DHBA molecule results in a change of the ligand field. Decrease in absorbance at 575 nm is at first observed. The formation of **2** is consistent with the formation and subsequent transformation (substitution) kinetics. Experiments conducted at various temperatures (279, 286, 290, and 295 K) yielded identical kinetic behavior.

**Figure 4:**
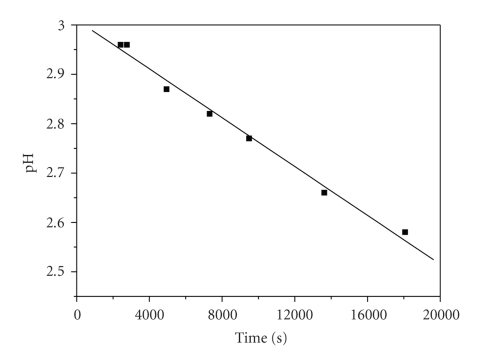
Typical pH versus time plot of 3,4-DHBA/Cr(III) mixtures at 313 K. Conditions: [3,4-DHBA]_0_ = 1.62 × 10^−2^ M, [Cr(III)]_0_ = 2.50 × 10^−3^ M, [KOH] = 4.00 × 10^−3^ M, I = 0.02 M.

However, the attacks by Cr(III) can take place only by releasing protons because the hydroxyls and the carboxylic groups are efficiently blocked (are protonated). This results in the pH decrease of the solution (Figure 4). Thus, the *k*_1_ pathway occurs by attack of Cr(H_2_O)_5_OH^2+^ at the carboxylic ligand group leading to a complex which, in acidic solutions, is protonated, **2**.

The kinetics of the first stage of the reaction (*k*_1(obs)_′) followed a first-order rate law, *k*_1(obs)_′ = *k*_0_ + *k*_1(obs)_[3, 4-DHBA]. The rate constant *k*_0_ corresponds to a reaction of Cr(III) when [3,4-DHBA] = 0.0, that is, to a reaction which does not involve the ligand. This may be any reaction of Cr(III) with KOH or with other Cr(III) ions, that is, an oligomerization reaction which always takes place in Cr(III) aqueous solutions. This does not affect the reactions and the mechanism of the reaction with 3,4-DHBA.

**Figure 5:**
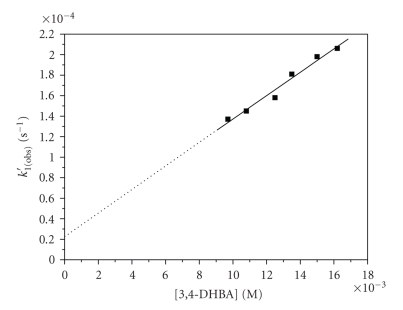
Dependence of *k*_1(obs)_′ on ligand concentration at 279 K and [Cr(III)] = 2.50 × 10^−3^ M, I = 0.02 M.

That is, in order to obtain information on how many ligands react in the first step, the dependence of *k*_1(obs)_′ on the ligand concentration was studied (Figure 5). In previous studies [24, 25], where the reactions of Cr(III) with 3,4-dihydroxyphenylpropionic acid (dihydrocaffeic acid) and 3,4-dihydroxyphenylpropenoic acid (caffeic acid) were investigated kinetically, the experimental data led to the conclusion that the reactive form of the metal ion is Cr(OH)^2+^ and the reactive form of the ligand is the neutral species.

A dissociative mechanism, *I*_*d*_ is expected for step 1 since, in the case of the conjugate base Cr(H_2_O)_5_OH^2+^, the dissociative mechanism *I*_*d*_ is favored [26]. This is because of the strong labilizing effect, which is induced by the coordinated OH^−^, presumably, on the trans H_2_O molecule. This leads to a 10^2^-10^3^ fold enhanced reaction rate for the hydroxy-aqua over the hexaaqua ion.

**Scheme 1:**
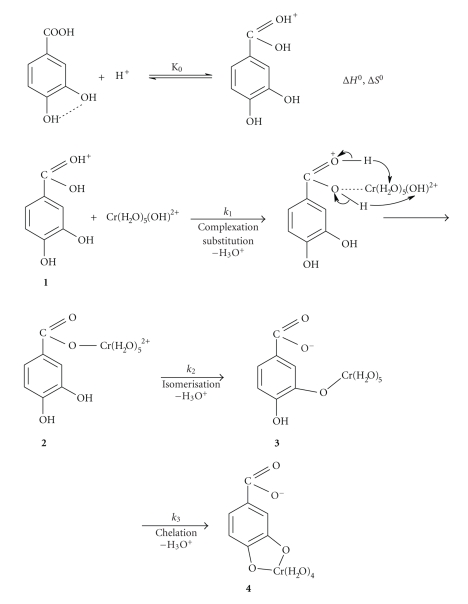


In conclusion, for the first attack, a reaction of Cr(OH)^2+^ can be proposed (Scheme 1). This would suggest an inverse dependence on [H^+^] since [Cr(OH)^2+^] = K_a_[Cr_(aq)_^3 +^]/[H^+^], which is not observed, implying that a protonation of the hydrogen-bonded ligand molecule exists; a breakout of the hydrogen bonding must take place before the attack by Cr(OH)^2+^. We suggest attack on the –OH of the protonated –COOH group (–HOC=OH^+^) since there the proton is loosely attached to oxygen due to the fact that withdrawing of negative charge by the H^+^ of the C=OH^+^ is taking place. The protonation of the –COOH group though in small extent makes the H of the OH more labile and so the attack by Cr(H_2_O)_5_(OH)^2+^ is easier. The small extent of the protonation of the –COOH group is the reason for the small yield of the reaction which is expressed by the small absorbance change Δ*A*.

Scheme 1 presents the mechanism proposed, according to the above experimental results.

Therefore, it holds that Rate = *k*_1_[Cr(OH)^2+^][3, 4-DHBAH^+^] = *k*_1_K_0_[3, 4-DHBA][H^+^](K_a_[Cr_(aq)_^3 +^]/[H^+^]) = *k*_1_K_0_K_a_[3, 4-DHBA][Cr_(aq)_^3 +^] = *k*_1(obs)_[3, 4-DHBA][Cr_(aq)_^3 +^], where *k*_1(obs)_ = *k*_1_K_0_K_a_.

**Figure 6:**
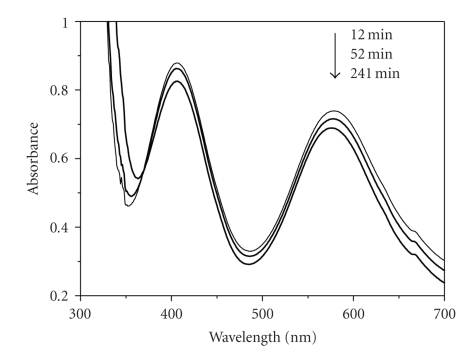
Typical UV-vis spectra of 3,4-DHBA/Cr(III) mixture at various times after mixing at 288 K. Conditions: [3,4-DHBA] = 6.80 × 10^−3^ M, [Cr(III)] = 3.75 × 10^−2^ M.

In Figure 6, spectra of the reaction mixture at 288 K are recorded at various times after mixing. The decrease in absorbance at short reaction times and the increase in longer reaction times suggest that the reaction is a multistep process. Spectra are shown only for the decrease in absorbance since at longer reaction time the increase in absorbance that follows would cause overlapping of the spectra.

The calculated negative value of Δ*S*_1(obs)_^*≠*^ would suggest an associative mechanism for the first stage of the reaction. Cr(H_2_O)_5_OH^2+^, however, as stated above, bears a dissociative, (*I*_*d*_), mechanism. This suggests, as presented above, that a complex reaction is taking place and composite activation parameters exist, that is, Δ*H*_1(obs)_^*≠*^ = Δ*H*_0_ + Δ*H*_*a*_ + Δ*H*_1_^*≠*^ and Δ*S*_1(obs)_^*≠*^ = Δ*S*_0_ + Δ*S*_*a*_ + Δ*S*_1_^*≠*^, where Δ*H*_0_ and Δ*S*_0_ correspond to an equilibrium established prior to step 1. Δ*H*_1_^*≠*^ and Δ*S*_1_^*≠*^ correspond to the first step (*k*_1_). Therefore, the resulting negative value of Δ*S* ^*≠*^ as well as the resulting value of Δ*H* ^*≠*^ do not correspond only to step 1 reaction which is actually taking place by an *I*_*d*_ mechanism due to the reactive species Cr(H_2_O)_5_OH^2+^.

Dependence on ligand and Cr(III) concentrations was studied in order to find if a second or third ligand molecule or Cr(III) species enters the coordination sphere of the already formed complex **2**. Therefore, the possibility of two consecutive steps involving transformations of **2** to **3** and of **3** to **4** $\left(2\overset{k_{2}}{\to }3\overset{k_{3}}{\to }4\right)$ is investigated.

The above scheme $2\overset{k_{2}}{\to }3\overset{k_{3}}{\to }4$ should give a biphasic reaction resembling a single-stage reaction when a polyfunctional compound reacts sequentially [20]. Thus, in our case, the polyfunctional ligand 3,4-DHBA reacting with Cr(III) gives the polyfunctional compound 2 which reacts sequentially and the biphasic reaction resembles a single-stage reaction giving a linear plot of ln(*A*_∞_ − *A*_*t*_) = *f *(*t*). This could also happen when *k*_3_ ≫ *k*_2_.

**Figure 7:**
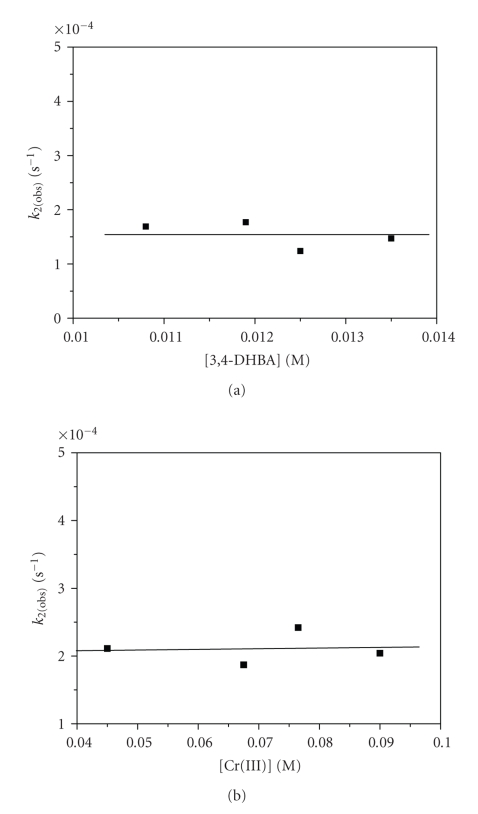
Dependence of *k*_2(obs)_ upon (a) ligand concentration at [Cr(III)] = 2.50 × 10^−3^ M, I = 0.02 M, 313 K and (b) Cr(III) concentration at [3,4-DHBA] = 7.45 × 10^−3^ M, I = 0.5 M, 318 K.

The experimental results (Figure 7) clearly demonstrate that the concentrations of 3,4-DHBA and Cr(III) have no effect on the observed rate constants *k*_2(obs)_ (≪*k*_3(obs)_) in the concentration range studied. However, study at higher concentrations was restricted due to the low solubility of 3,4-DHBA in weak acidic aqueous solutions. Values of *k*_2(obs)_ (≪*k*_3(obs)_) are presented in Table 1 and the corresponding Δ*H*_2(obs)_^*≠*^, Δ*S*_2(obs)_^*≠*^ values are listed in Table 2.

The fact that the second and third steps (*k*_2_, *k*_3_) were found to be independent both on ligand and Cr(III) concentrations, that is, the rate exhibits a first-order dependence on the product of step 1, suggests that transformations are taking place within the already formed complex, **2**.

**Scheme 2:**
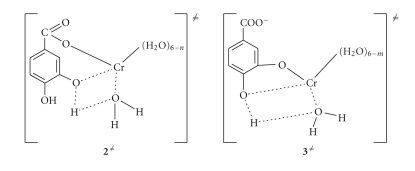
Activated complexes **2** ^*≠*^ and **3** ^*≠*^.

The activation parameters deduced from the temperature-dependence experiments may be used for proposing structures of the activated complexes (Scheme 2), and the taking place mechanism.

The negative value of Δ*S*_2_^*≠*^, the independence of *k*_2(obs)_ (*k*_2_ ≪ *k*_3_) on both ligand and Cr(III) concentrations, and the increase in absorbance (i.e., of the extinction coefficients) led to the assignment of the observed transformations as *associatively activated substitution* of water molecules from the Cr(III) coordination sphere by 3,4-DHBA through isomerization and chelation in two consecutive steps ($2\overset{k_{2}}{\to }3\overset{k_{3}}{\to }4$). The pH decrease suggests a concomitant proton release as proposed in the mechanism (Scheme 1). Scheme 1 shows that **2**, the first formed species, undergoes isomerization to give another oxygen-bonded species, **3**. Complex **3** chelates in a *k*_3_ step, (*k*_2_ ≪ *k*_3_) chelation, to produce **4**, the final chelated complex [Cr(3,4-DHBA_−3H_)(H_2_O)_4_]. Isolation in the solid form of the final product gives a compound the elemental analyses of which (15.4% C and 3.7% H) correspond to the formula [Cr(3,4-DHBA_−3H_)(H_2_O)_4_]·2KNO_3_·4H_2_O, for which the calculated percentages for C and H are 15.3% and 3.5%, respectively.

Alternatively, the first attack could occur at the hydroxyl group (step *k*_1_) and a following chelation could account for a second step (*k*_2_) only. We prefer though the already suggested mechanism which is in accord with the mechanisms of the reactions of Cr(III) with 3,4-dihydroxyphenylpropionic acid and 3,4-dihydroxyphenylpropenoic acid where the three stages (i.e., complexation, isomerization, and chelation) were kinetically distinguishable [24, 25]. In our present case, three steps are also suggested because of the necessity for isomerization prior to chelation. Chelation between the groups carboxylic and phenolic is unfavorable due to the formation of seven-membered ring. We exclude the possibility of a reversible reaction, where the final absorbance could correspond to Cr(III) and complex (in equilibrium) resulting in *k*_obs_ = *k*_*f*_ + *k*_*r*_ based on the inertness of the various species (small values of reaction rates) and the existence of further reactions which, then, would shift an equilibrium well to the right.

Another possibility is attack at the carboxylic group and subsequent chelation at the same group through the oxygens. We are discarding this alternative because a four-membered ring would result if chelation at the carboxylic group took place whereas a more preferable situation, that is, a five-membered ring is formed according to the suggested mechanism.

Since Cr, Mo, and W are in the same group of the periodic table, the following comparison can be made: [Cr(H_2_O)_5_(OH)]^2+^ in our system reacts with a rate constant two orders of magnitude slower than that of [W_3_O_4_(H_2_O)_9_]^4+^ and three orders of magnitude slower than that of [Mo_3_O_4_(H_2_O)_9_]^4+^ indicating an extremely less labile species [27]. The comparison is made for substitution reactions of the conjugate base forms of the above aqua ions.

### 3.2. Structure of the complexes—mode of binding

In the UV-vis spectra, the changes are caused only by the change of the ligand field. Since oxidation does not take place under the experimental conditions, only a shift of the maximum and disappearance of the shoulder due to the high absorbtivities in the UV region of the produced complexes take place.

A 1 : 1 stoichiometry for the reaction of Cr(III) with 3,4-DHBA is proposed because of the observed *k*_1_ dependence on ligand concentration. The elemental analyses of the isolated final product also suggest a 1 : 1 stoichiometry. The consecutive isomerization and chelation reactions taking place in the Cr(III) center do not cause any change in stoichiometry.

The proposed structures of the activated complexes **2** ^*≠*^ and **3** ^*≠*^ are given in Scheme 2. Associative mechanism has been supported to operate in reactions of Cr(III) [18, 28, 29]. In the above suggested mechanism, the phenolic groups act as internal attacking groups to the H_2_O molecules of the Cr(III) coordination sphere supplying thus a proton, which is then released as H_3_O^+^ (Scheme 1).

The driving force for the production of the final chelated product **4** must be the most stabilized chelated form compared to forms **2** or **3**.

The suggested catecholic mode of binding was also found to operate in the coordination complexes of 3,4-dihydroxyphenylpropionic acid (dihydrocaffeic acid) and 3,4-dihydroxyphenylpropenoic acid (caffeic acid) with Cr(III) [24, 25] as already mentioned. This type of binding was also reported for complexes of dihydrocaffeic, caffeic, and ferulic acids with Co(II), Ni(II), Cu(II), Fe(III), Mn(II), Mn(III), V(V), V(IV,V), and Zn(II) [22, 23, 30–32]. Catecholic type of coordination was also suggested for the Fe(III)-2,3-DHBA complex [33, 34].

## 4. CONCLUSIONS

In the present study, the reaction between Cr(III) and 3,4-DHBA in weak acidic aqueous solutions was investigated. The experimental results are consistent with a three-step mechanism in which an initial attack (step 1) between the acid molecule (ligand) and the Cr(H_2_O)_5_OH^2+^ complex giving a carboxylate bound Cr(III), **2**, is followed by two consecutive kinetically indistinguishable nonligand and non-Cr(III) dependent steps. The two consecutive steps are assigned as isomerization and chelation step (steps 2 and 3). The reactions are followed by a pH decrease because proton release is taking place, according to an associative mode of activation (steps 2 and 3).

The negative value of the entropy of activation of step 2, the independence on ligand and Cr(III) concentrations, the increase of the extinction coefficients, and the pH decrease due to release of protons upon complexation led to the proposed mechanism (Scheme 1). The observed transformations were assigned as substitution of water molecules from the coordination sphere of Cr(III) by the ligand through complexation (step 1) following an *I*_*d*_ mechanism, isomerization, and chelation in two consecutive kinetically indistinguishable steps supported to follow associative mechanisms (step 2 and step 3).

## Figures and Tables

**Table 1:** Values of *k*_1(obs)_ and *k*_2_ (*k*_2_ ≪ *k*_3_) at various temperatures.

Temperature (K)	Rate constants	
	*k*_1(obs)_ × 10^2^ (M^−1^ s^−1^)	*k*_2_ × 10^4^ (s^−1^)
279	1.10	—
286	2.52	—
290	3.15	—
295	3.74	—
303	—	0.91
309	—	1.35
313	—	1.54
318	—	2.11
323	—	3.11

**Table 2:** Activation parameters for steps 1 (*k*_1(obs)_) and 2 (*k*_2_ ≪ *k*_3_).

Δ*H*_1(obs)_^*≠*^ (kJ mol^−1^)	Δ*S*_1(obs)_^*≠*^ (J mol^−1^ K^−1^)	Δ*H*_2(obs)_^*≠*^ (kJ mol^−1^)	Δ*S*_2(obs)_^*≠*^ (J mol^−1^ K^−1^)
51.2 ± 11.5	−97.3 ± 28.9	44.5 ± 5.0	−175.8 ± 70.3
